# Robo2 is required for Slit-mediated intraretinal axon guidance

**DOI:** 10.1016/j.ydbio.2009.09.034

**Published:** 2009-11-15

**Authors:** Hannah Thompson, William Andrews, John G. Parnavelas, Lynda Erskine

**Affiliations:** a Institute of Ophthalmology, University College London, Bath Street, London, EC1V 9EL, England, UK; b Department of Veterinary Basic Sciences, Royal Veterinary College, Royal College Street, London, NW1 0TU, England, UK; c Department of Cell and Developmental Biology, University College London, Gower Street, London, WC1E 6BT, England, UK; d School of Medical Sciences, Institute of Medical Sciences, University of Aberdeen, Foresterhill, Aberdeen, AB25 2ZD, Scotland, UK

**Keywords:** Robo, Slit, Axon guidance, Retinal ganglion cell, Growth cone, Retina, Visual system, Development

## Abstract

The developing optic pathway has proven one of the most informative model systems for studying mechanisms of axon guidance. The first step in this process is the directed extension of retinal ganglion cell (RGC) axons within the optic fibre layer (OFL) of the retina towards their exit point from the eye, the optic disc. Previously, we have shown that the inhibitory guidance molecules, Slit1 and Slit2, regulate two distinct aspects of intraretinal axon guidance in a region-specific manner. Using knockout mice, we have found that both of these guidance activities are mediated via Robo2. Of the four vertebrate Robos, only Robo1 and Robo2 are expressed by RGCs. In mice lacking *robo1* intraretinal axon guidance occurs normally. However, in mice lacking *robo2* RGC axons make qualitatively and quantitatively identical intraretinal pathfinding errors to those reported previously in Slit mutants. This demonstrates clearly that, as in other regions of the optic pathway, Robo2 is the major receptor required for intraretinal axon guidance. Furthermore, the results suggest strongly that redundancy with other guidance signals rather than different receptor utilisation is the most likely explanation for the regional specificity of Slit function during intraretinal axon pathfinding.

## Introduction

The first step in the formation of functional visual connections is the guidance of retinal ganglion cell (RGC) axons towards the optic disc, their exit point from the eye. Intraretinal axon guidance involves 3 distinct process: growth of RGC axons into the optic fibre layer (OFL) at the inner surface of the retina, directed extension within the OFL towards the optic disc and exit from the eye (reviewed by Erskine and Herrera, 2007; Bao, 2008; Erskine and Thompson, 2008).

Several molecules have been implicated in directing distinct aspects of intraretinal axon guidance. In rodents, a central–peripheral wave of chondroitin sulphate proteoglycan (CSPG) and Slit2 directs the initial outgrowth of axons away from the retinal periphery (Brittis et al., 1992; Brittis and Silver, 1995; Thompson et al., 2006a). More centrally, EphB2/B3, BMP (bone morphogenic protein) receptor 1B and Netrin1 are required for targeting to and entry into the optic disc (Deiner et al., 1997; Birgbauer et al., 2000; Liu et al., 2003). In chicks, graded expression of Sonic hedgehog across the retina plays a role in directing the central–peripheral growth of the axons (Kolpak et al., 2005). A number of short-range signals in the form of cell adhesion molecules also have been implicated, including NCAM (neural cell adhesion molecule), NrCAM, NCAM associated PSA, L1 and Neurolin (also called DM-GRASP/BEN/SC1; Pollerberg and Beck-Sickinger, 1993; Brittis et al., 1995; Ott et al., 1998; Monnier et al., 2001; Avci et al., 2004; Weiner et al., 2004; Zelina et al., 2005).

Slit and Robo, discovered in *Drosophila* as key regulators of commissural axon guidance (Rothberg et al., 1990; Seeger et al., 1993; Kidd et al., 1998), are essential for RGC axon pathfinding in the vertebrate visual system (Erskine et al., 2000; Niclou et al., 2000; Ringstedt et al., 2000; Fricke et al., 2001; Plump et al., 2002; Thompson et al., 2006a,b; Plachez et al., 2008). There are three vertebrate Slits and four Robos (Kidd et al., 1998, 1999; Brose et al., 1999; Yuan et al., 1999; Huminiecki and Bicknell, 2000). Recently, we have shown that Slits regulate two distinct aspects of intraretinal axon guidance in a spatially restricted manner. Within dorsal retina exclusively, Slits control the initial polarity of RGC axon outgrowth and prevent a subset of RGC axons located predominately in the ventral retina from straying away from the OFL (Thompson et al., 2006a). Since vertebrates have multiple Robos, at least two of which are expressed in the developing retina (Erskine et al., 2000; Niclou et al., 2000; Ringstedt et al., 2000; Plachez et al., 2008), could distinct receptors mediate these different aspects of intraretinal axon guidance? Using in situ hybridisation and immunohistochemistry, we have confirmed that Robo1 and Robo2 are the only Robo family members expressed by RGC axons. To determine whether these receptors contribute differentially to intraretinal axon guidance, we examined retinas from *robo1*- and *robo2*-deficient mouse embryos for RGC axon pathfinding defects. The results demonstrate clearly that Robo2 regulates both aspects of Slit-mediated intraretinal axon pathfinding. This suggests strongly that redundant interactions with other guidance signals, rather than different receptor utilisation, underlie the distinct requirements of Slits in dorsal and ventral retina.

## Materials and methods

### Embryos

Experiments were performed using wild-type C57bl/6J, *robo-*deficient (Lu et al., 2007; Andrews et al., 2008) or *slit*-deficient mice (Plump et al., 2002) maintained in in-house, timed-pregnancy breeding colonies. Noon on the day on which a plug was found was considered embryonic day 0.5 (E0.5). Pregnant mothers were killed using a rising gradient of CO_2_ and the embryos removed by caesarean section. Embryos were genotyped by PCR as described previously (Plump et al., 2002; Lu et al., 2007; Andrews et al., 2008) and fixed overnight in 4% paraformaldehyde in phosphate buffered saline (PBS).

### In situ hybridisation

The *robo1-3* templates for riboprobe synthesis have been described previously (Brose et al., 1999; Sabatier et al., 2004). The *robo4* template was generated by subcloning a *Xho*I and *Hin*dIII fragment of mouse EST 3978776 (Invitrogen, Paisley, UK), corresponding to nucleotides 187-1427 of mouse *robo4*, into pBluescript KS+. In situ hybridisation, using digoxigenin-labelled riboprobes was performed on 100-μm coronal sections of E16.5 wild-type embryonic heads as described previously (Erskine et al., 2000). Briefly, sections were dehydrated and rehydrated in 25–100% methanol in PBT (PBS + 0.1% Tween-20), bleached with 6% H_2_O_2_ (Sigma-Aldrich, Dorset, UK) in PBT for 1 h, treated with 5 μg/ml Proteinase K (Sigma-Aldrich) in PBT for 10 min, followed by postfixation with 4% PFA in PBT. The sections were incubated at 65 °C in hybridisation buffer (50% Formamide, 5× SSC, 50 μg/ml tRNA, 1% SDS, 50 μg/ml Heparin) for 1 h, followed by hybridisation overnight at 65 °C with probes diluted 1:100 in hybridisation buffer. The sections were washed three times with 50% formamide, 5× SSC, 1% SDS at 65 °C then with 50% formamide, 2× SSC at 60 °C, blocked with 10% sheep serum/TBST (TBS + 1% Tween-20) and incubated overnight in anti-DIG-alkaline phosphatase (AP) antibody (Roche Diagnostics, Lewes, UK) diluted 1:2000 in 1% sheep serum/TBST. To activate the colour reaction, the sections were washed extensively with TBST and the AP activity detected using NBT (337.5 μg/ml, Sigma-Aldrich) and BCIP (175 μg/ml; Sigma-Aldrich) in NTMT (100 mM NaCl, 100 mM Tris–HCl, pH 9.5, 50 mM MgCl2, 1% Tween-20). Sections were mounted in 90% glycerol/PBS.

### Immunostaining and DiI labelling

For anti-Robo immunohistochemistry, E16.5 mouse brains were fixed with 4% PFA in PBS for 30 min, cyrosectioned at 15 μm, treated with 0.3% H_2_O_2_ for 45 min and washed 3 times in PBS. Sections were blocked in TSA tetramethylrhodamine system blocking reagent (Perkin-Elmer, Bucks, UK)/0.3% Triton for 2 h before incubating in anti-Robo antibodies (R&D systems, Abingdon, UK) in the same blocking solution for two nights, followed by biotinylated rabbit anti-goat IgG (Vector Labs, Peterborough, UK; 1:200) in the same blocking solution for 2 h. The signal was amplified using avidin-biotinylated enzyme complex from the Vectorstain Elite ABC kit (Vector Labs) for 2 h. The sections were washed five times in PBS and the colour developed using DAB (Sigma-Aldrich). The sections were dehydrated in ethanol (75–100%), washed in Histoclear (Fisher Scientific, UK) for 5 min and mounted with DPX. Control sections from which the primary antibody was omitted showed no staining (data not shown).

For immunohistochemistry on whole retinas, prior to removing the retinas, a cut was made in the nasal pole to enable orientation and the lens removed. Retinas were blocked with 10% goat serum/0.2% Triton X-100/PBS and incubated for two days in antibodies against neuron-specific β-tubulin (TUJ1; Cambridge Bioscience Ltd., Cambridge, UK; 1:1000), Islet1/2 (39.4D5; Developmental Studies Hybridoma Bank (DSHB; 1:50), AP2α (3b5; DSHB; 1:50), Pax6 (DSHB; 1:400), phosphohistone-H3 (Millipore, Herts, UK; 1:100) or Brn3a (Millipore; 1:100) in blocking solution followed by a 2-h incubation in the appropriate secondary antibody (AlexaFluor-594 goat anti-mouse IgG_2b_ (Invitrogen Ltd.; 1:250), AlexaFluor-488 goat anti-mouse IgG_1_ (Invitrogen Ltd.; 1:250), AlexaFluor-488 goat anti-mouse IgG (Invitrogen Ltd; 1:250) or Cy3-goat anti-rabbit IgG (Jackson ImmunoResearch, Soham, UK; 1:1000)). Labelled retinas were flat mounted, OFL-side up, or embedded in 3% agarose, sectioned at 100 μm on a vibratome and mounted using ProLong Gold (Invitrogen Ltd) or Vectashield (Vector labs). Control preparations from which the primary antibody was omitted showed no staining (data not shown).

To examine the growth of RGC axons out of the optic disc, a small crystal of DiI (Invitrogen Ltd) was placed in the peripheral region of either the dorsal or ventral retina of E16.5 wild-type, *slit1/2-*, *robo-1* or *robo2-*deficient retinas. The tissue was incubated in PBS at 37 °C for 6 h then flat mounted using Vectashield. To label retina–retina axons, a crystal of DiI was placed over the optic disc of one retina in E16.5 wild-type or *robo2*-deficient embryos and the heads left in 4% PFA in PBS + 0.02% azide at room temperature for 6–8 weeks. The contralateral retina was dissected out and either flat mounted or sectioned at 100 μm and mounted in Vectashield.

Labelled sections and retinas were photographed using an Olympus BX50 microscope and a Nikon DXM1200 digital camera with ACT-1 software or using a Zeiss LSM 510 confocal microscope. Images were prepared using Adobe Photoshop.

## Results

### Robo receptors are expressed differentially by RGCs

**Fig. 1:**
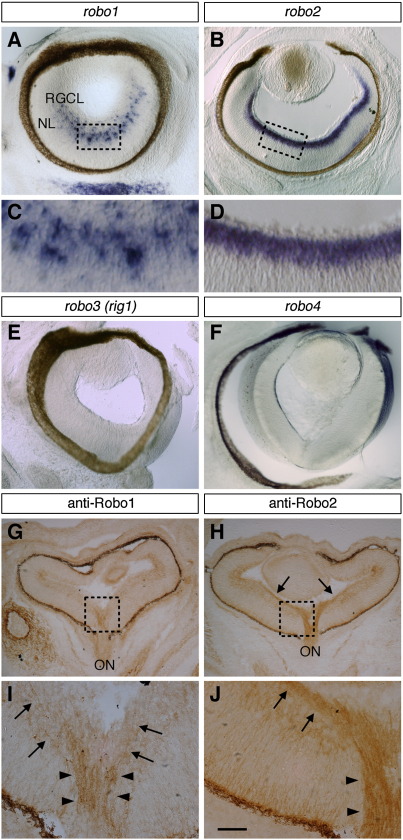
Robo1 and Robo2 are expressed by RGCs. (A–F) Coronal sections of E16.5 wild-type retinas stained by *in situ* hybridisation for *robo1* (A, C), *robo2* (B, D), *robo3 (rig1;* E) or *robo4* (F). *robo1* and *robo2* are expressed in the RGC layer whereas no expression of *robo3* and *robo4* was detected in the retina. The boxed regions in A and B are shown at higher power in C and D, respectively. (G, H) Coronal sections of E16.5 wild-type retinas stained with antibodies against Robo1 (G) or Robo2 (H). (I, J) Higher power images of the boxed regions in G and H, respectively. Robo1 and Robo2 are expressed by RGC axons in the optic fibre layer (arrows) and optic nerve (arrowheads). RGCL, retinal ganglion cell layer; NL, neuroblastic layer; ON, optic nerve. Scale bar, 200 μm (A, B, E–H) and 40 μm (C, D, I, J).

Using in situ hybridisation, we previously found that *robo1* and *robo2* are expressed by cells in the RGC layer of the retina suggesting expression by RGCs (Erskine et al., 2000). Here we noted that at E16.5 *robo1* is restricted to a subset of cells in the RGC layer (Figs. 1A and C) whereas *robo2* is expressed by most, if not all, such cells (Figs. 1B and D). In addition, we confirmed that Robo1 and Robo2 proteins are expressed by RGCs. Strong expression of both Robo1 and Robo2 proteins was detected in the RGC layer and on RGC axons in the OFL, optic nerve head as well as other regions of the developing optic pathway (Figs. 1G–J; data not shown). In agreement with the in situ hybridisation results, Robo1 protein is present on a small subset of axons whereas Robo2 protein is more widely expressed. Robo1 and Robo2 are the only Robos expressed by RGCs as no expression of the other two known *robos*, *robo3/rig1* and *robo4*, was detected within the retina (Figs. 1E and F).

### Robo2 restricts RGC axons to the OFL

**Fig. 2:**
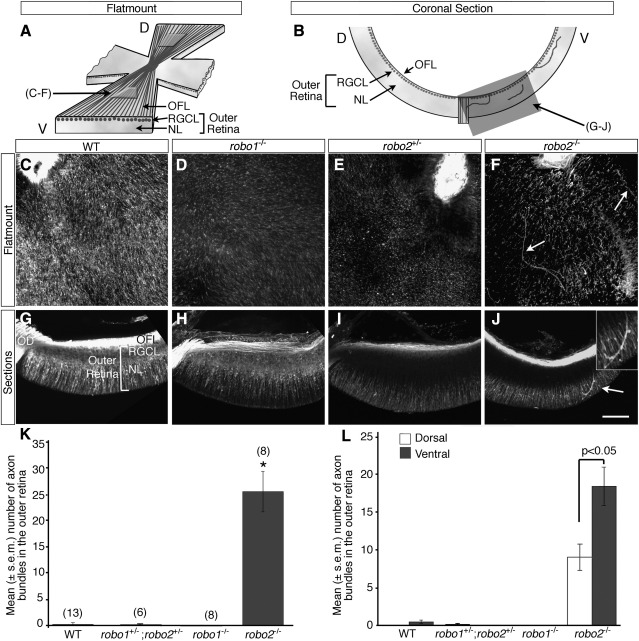
Robo2 helps restrict RGC axons to the optic fibre layer. (A, B) Schematic diagrams illustrating the regions of the retinas imaged. (C–F) Confocal images taken at the level of the neuroblastic layer of flat-mounted E16.5 retinas stained with an anti-β-tubulin antibody. The optic disc is located towards the top of each picture. (G–J) Coronal sections of anti-β-tubulin stained E16.5 retinas. In each image the optic disc is located towards the left-hand side. In wild-type (C, G), *robo1^−^*^/*−*^ (D, H) and *robo2*^+/*−*^ (E, I) retinas, RGC axons are restricted to the optic fibre layer. In *robo2*-deficient retinas (F, J), large bundles of axons (arrows) are located within the outer retina (retinal ganglion cell and neuroblastic layers). Inset in J shows the ectopic axons at higher magnification. D, dorsal; NL, neuroblastic layer; OD, optic disc; OFL, optic fibre layer; RGCL, retinal ganglion cell layer; V, ventral. Scale bar, 100 μm (C–J). (K) Mean ± SEM number of axon bundles located within the outer retina of wild-type, *robo1*^+/*−*^;*robo2*^+/*−*^, *robo1^−^*^/*−*^ and *robo2^−^*^/*−*^ retinas. ⁎*p* < 0.001 compared with wild-type. Numbers above bars = numbers analysed. (L) Mean ± SEM number of axons bundles in the outer layers of dorsal (white bars) or ventral (grey bars) retina of wild-type, *robo1*^+/*−*^;*robo2*^+/*−*^, *robo1^−^*^/*−*^ and *robo2^−^*^/*−*^ embryos. In *robo2^−^*^/*−*^ retinas, significantly more ectopic bundles of axons are found within the outer layers of ventral versus dorsal retina.

Slits have been shown previously to control two distinct aspects of intraretinal axon pathfinding in a regional-specific manner (Thompson et al., 2006a). Since RGC axons express both Robo1 and Robo2, one explanation for these distinct guidance aspects of Slits is that they are mediated by different Robo receptors. To test this notion we examined intraretinal axon pathfinding in *robo1-* or *robo2*-deficient mice (Lu et al., 2007; Andrews et al., 2008). Retinas from wild-type, *robo1-* or *robo2-*deficient mice (E14.5-E18.5) were stained with an anti-β-tubulin antibody and viewed with a confocal microscope in flat mounts or coronal sections (Figs. 2A–J). In wild-type retinas, the vast majority of RGC axons were restricted to the OFL (Figs. 2C, G, K, L). In only 1 of 13 wild-type retinas were any axons seen within the outer retinal layers (RGC layer and neuroblastic layer). The same was seen for *robo1^+/−^* (1/10), *robo1^−/−^* (0/8; Figs. 2D, H, K, L), *robo2^+/−^* (0/5; Figs. 2E and I) and *robo1*^+/*−*^;*robo2*^+/*−*^ (1/6; Figs. 2K and L) retinas. By contrast, in *robo2^−^*^/*−*^ embryos, a significant number of RGC axons grew aberrantly away from the OFL into the outer retina (8/8; Figs. 2F, J–L). These ectopically located axons formed highly fasciculated bundles and appeared to extend in the overall direction of the optic disc. An average of 25 bundles of axons was seen in the outer layers of each *robo2^−^*^/*−*^ retina. These ectopic axon bundles were not distributed uniformly throughout the retina—over twice as many axon bundles extended through the outer layers of ventral than dorsal retina (Fig. 2L). Both the number and distribution of these ectopic axon bundles was identical to that seen in *slit1/2* double mutants (Thompson et al., 2006a). We were unable to generate any double *robo1^−^*^/*−*^;*robo2^−^*^/*−*^ embryos despite extensive efforts and therefore cannot exclude definitively that Robo1, by functioning redundantly with Robo2, may make a minor contribution to this aspect of intraretinal axon pathfinding. However, since the guidance errors in the *robo2*-single mutants are qualitatively and quantitatively identical to those seen in the *slit* mutants (Thompson et al., 2006a), this suggests strongly that Robo2 is the only Robo required for the Slit-mediated restriction of RGC axons to the OFL.

### Ectopic axons in the outer layers of robo2-deficeint retinas are not misguided axons from the contralateral eye

**Fig. 3:**
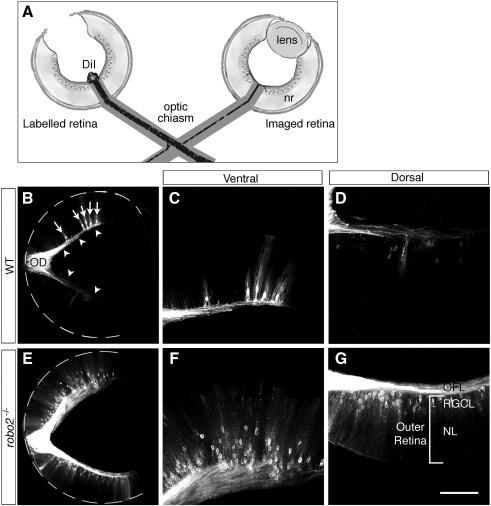
Ectopic axons in the outer retina of *robo2*-deficient embryos are not retina–retina axons originating in the contralateral eye. (A) Schematic diagram illustrating retina–retina labelling of RGC axons using DiI. A crystal of DiI is placed on the optic disc of one retina and the contralateral eye is imaged. nr, neural retina. (B–G) Coronal sections of wild-type (B–D) and *robo2-*deficient (E–G) retinas DiI-labelled from the contralateral eye. (B) In wild-type mice, a small number of axons project to the contralateral retina where they terminate with a higher frequency in ventral (C) than dorsal retina (D). Axons that originate in the imaged eye and project to the contralateral (labelled) eye also are labelled. Arrows indicate the cell bodies of labelled RGCs; arrowheads, labelled RGC axons in the optic fibre layer. (E) In *robo2*-deficient mice significantly more RGC axons project to the contralateral eye. As in wild-type mice, these retina–retina axons are restricted to the optic fibre layer of the retina and terminate more frequently in ventral (F) than dorsal (G) retina. More labelled cell bodies of axons that originate in the imaged eye and project to the contralateral (labelled) eye also are present. NL, neuroblastic layer; OD, optic disc; OFL, optic fibre layer; RGCL, retinal ganglion cell layer; WT, wild-type. Scale bar, 200 μm (B, E) and 100 μm (C, D, F, G).

In wild-type mice, a small proportion of RGC axons project into the contralateral optic nerve and terminate predominately in the ventral part of the contralateral retina (Figs. 3B–D; Plump et al., 2002). This ventral bias is the same distribution pattern as occurs for the ectopic RGC axons in the outer layers of the *slit1/2-* and *robo2*-deficient retinas (Fig. 2; Thompson et al., 2006a). To investigate if the ectopic RGC axons located in the outer layers of the *robo2*-deficient retinas are aberrant retina–retina axons, we labelled the RGC axons from one eye of E16.5 wild-type or *robo2*-deficient embryos with DiI and, after allowing time for the dye to transport, examined the localisation of the labelled axons in the contralateral eye (Fig. 3A). In wild-type and *robo2^+/−^* retinas, a small number of RGC axons projected to the contralateral retina. These retina–retina axons were restricted to the OFL and found in higher numbers in ventral than dorsal retina (Figs. 3B–D, Table 1). In *robo2*-deficient mice many more RGC axons than normal project into the contralateral optic nerve (Plachez et al., 2008). This was reflected by an increased number of labelled axons within the contralateral retina (Fig. 3E). As in wild-type retinas, a greater proportion of these retina–retina axons terminated in ventral than dorsal retina (Figs. 3F and G). In the vast majority of cases, these axons grew into and were restricted to the OFL (Figs. 3E–G; Table 1). In only 1/6 *robo2*-deficient retinas did a small number of retina–retina axons extend aberrantly through the outer layers of the contralateral retina. Furthermore, the number (3) of these ectopic retina–retina axon bundles was significantly smaller than that seen following labelling of the entire optic projection (25 ± 4). This suggests strongly that in the absence of *robo2*, the vast majority of RGC axons located in the outer layers of the retina are not misguided retina–retina axons but the result of pathfinding errors made by RGC axons prior to exiting the eye.

### Robo2 regulates the initial polarity of RGC axon outgrowth

**Fig. 4:**
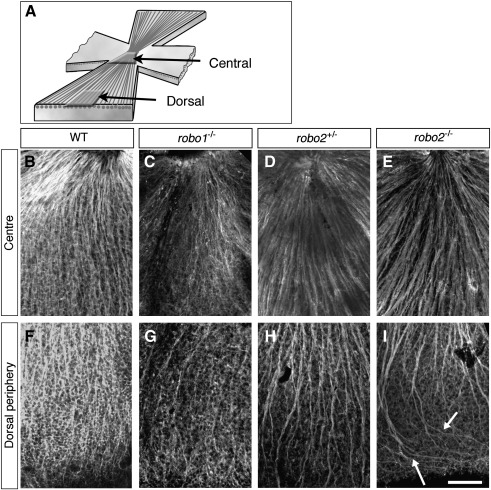
Robo2 helps control the initial direction of RGC axon outgrowth. (A) Schematic diagram illustrating the regions of the flat-mounted retinas imaged. (B–I) Confocal images taken at the level of the optic fibre layer of E16.5 retinas stained with an anti-β-tubulin antibody. In each image, the direction of the optic disc is towards the top of the picture. In wild-type (B, F), *robo1^−^*^/*−*^ (C, G) and *robo2*^+/*−*^ (D, H) retinas, RGC axons extend directly towards the optic disc. In *robo2^−^*^/*−*^ (E, I) retinas, RGC axons within the peripheral (I) but not central (E) region of the dorsal retina extend aberrantly away from the optic disc (arrows). WT, wild-type. Scale bar, 100 μm (B–I).

Slits, possibly originating from the lens, help control the initial polarity of RGC axon outgrowth within the OFL itself (Thompson et al., 2006a). We have found that Robo2 also is the major receptor required for this guidance activity (Fig. 4). In wild-type retinas, RGC axon outgrowth is highly polarised and extends in a directed, radial fashion straight towards the optic disc (Figs. 4B and F). In retinas from *robo1^+/−^* (*n* = 9), *robo1^−/−^* (*n* = 8; Figs. 4C and G), *robo2^+/−^* (*n* = 5; Figs. 4D and H) and *robo1*^+/*−*^;*robo2*^+/*−*^ (*n* = 6) embryos RGC axon guidance within the OFL occurred normally. However, in mice lacking *robo2* (*n* = 12), the initial polarity of RGC axon extension was perturbed (Fig. 4I). Rather than extending directly towards the optic disc, many axons exhibited abnormal looped and curved morphologies that, in some instances, even extended parallel to the outer surface of the retina. These guidance errors occurred specifically in the peripheral quarter of the retina, a region that contains the axons from the most recently differentiated RGCs. Guidance of more mature RGC axons located within central retina occurred normally (Fig. 4E). Again, these guidance errors were identical to those seen in the absence of Slits (Thompson et al., 2006a). Furthermore, in both *robo2-* and *slit-*deficient retinas the initial polarity of outgrowth from dorsal RGCs was affected exclusively. In retinas of either genotype, RGC axon outgrowth within the OFL of ventral retina was indistinguishable from wild-type (data not shown; Thompson et al., 2006a). Since we were unable to generate *robo1/2*-double mutants, it remains possible that Robo1 may make a minor contribution to controlling the initial polarity of RGC axon outgrowth. However, the identical phenotypes of the *robo2*- and *slit*-deficient mice suggest this is unlikely to be the case.

### Slit and Robo are not required for entry into the optic nerve

**Fig. 5:**
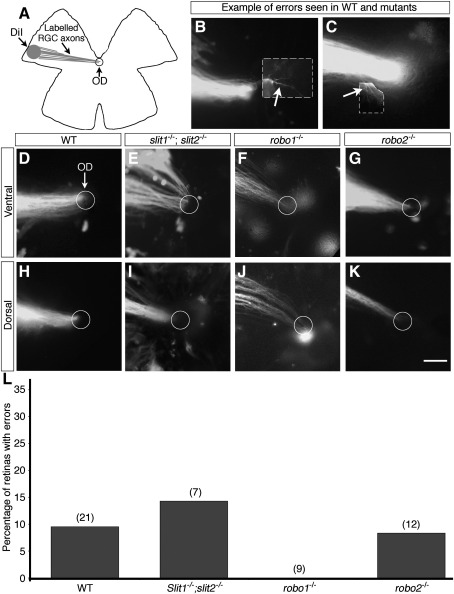
Slit–Robo signalling is not required for RGC axons to exit the eye. (A) Schematic diagram illustrating the method used to label small subsets of RGC axons with DiI. Small crystals of DiI were placed in the peripheral region of either dorsal or ventral retina. (B–K) Images of labelled ventral (D–G) or dorsal (H–K) RGC axons in the region of the optic disc (circle) in wild-type (B, C, D, H), *slit1^−/−;^;slit2^−/−^* (E, I) *robo1^−/−^* (F, J) or *robo2^−^*^/*−*^ (G, K) embryos. In retinas of each genotype, the vast majority of axons extend towards the optic disc and exit the eye normally. In only a small number of cases were minor targeting errors observed (B, C). OD, optic disc; WT, wild-type. Scale bar, 50 μm (B–C) and 100 μm (D–K). (L) Quantification of the percentage of wild-type (WT), *slit1^−/−^;slit2^−/−^ robo1^−/−^* and *robo2^−/−^* retinas with optic disc targeting errors. Numbers above bars = numbers analysed.

A number of axon guidance molecules have been found to be important for entry into the optic nerve and exit from the eye (Birgbauer et al., 2000; Deiner et al., 1997; Liu et al., 2003; Oster et al., 2003). To determine if Slit and Robo also are required for this process, subsets of RGC axons were visualised using focal DiI. Small crystals of DiI were placed in either the dorsal or ventral retina (Fig. 5A) in wild-type, *slit1/2-, robo1-* or *robo2-*deficient mice and the path of the labelled axons examined in flat mount preparations. In wild-type retinas, RGC axons extended as a tightly grouped bundle in a highly directed manner towards the central retina where they converged on the optic disc and exited the eye (Figs. 5D and H). In a small percentage of wild-type retinas (2/21; Figs. 5B and C) a few RGC axons bypassed the optic disc and extended into the contralateral retina. The length of these misprojecting axons was less than 1000 μm and classified as the less severe, type 2 errors (Birgbauer et al., 2000). In *slit1/2-*deficient (*n* = 7; Figs. 5E and I), *robo1-*deficient (*n* = 9; Figs. 5F and J) and *robo2*-deficient (*n* = 12; Figs. 5G and K) retinas, no increase in optic disc targeting errors occurred. In retinas of each genotype, the majority of RGC axons originating from either dorsal or ventral retina extended normally out of the eye. In only a small number of cases (1/7, *slit1/2-*deficient; 0/9, *robo1-*deficient; 1/12, *robo2-*deficient) were RGC axons that bypassed the optic disc (Fig. 5L). The incidence and severity of these targeting errors were indistinguishable from that seen in wild-type retinas. This demonstrated that Slit–Robo signalling is required exclusively for RGC axon guidance within the peripheral retina and not for growth out of the eye.

### Retinal morphology is normal in robo2-deficient embryos

**Fig. 6:**
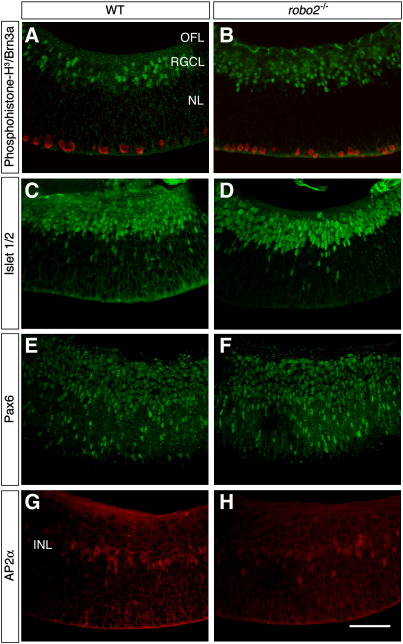
Retinal morphology is normal in *robo2*-deficient retinas. Coronal sections of E16.5 wild-type (A C, E, G) and *robo2^−^*^/*−*^ (B, D, F, H) retinas labelled with antibodies against phosphohistone-H^3^ (red) and Brn3a (green; A, B), Islet 1/2 (C, D), Pax6 (E, F) or AP2α (G, H). In each image the direction of the optic disc is on the left. In *robo2*-deficient retinas mitotic cells (B) and differentiated RGCs, amacrine cells and bipolar cells (B, D, F, H) are arrayed similar to wild-type (A, C, E, G). INL, inner nuclear layer; NL, neuroblastic layer; OFL, optic fibre layer; RGCL, retinal ganglion cell layer; WT, wild-type. Scale bar, 50 μm.

One explanation for the intraretinal guidance errors that occurs in the absence of *robo2* is that they occur secondary to changes in the structure or organisation of the retina. We do not believe this is the case. In *robo2-*deficient embryos, the overall size and shape of the retina appeared normal (data not shown). Using a number of cell-type-specific antibodies, we have also found that overall organisation and lamination of the retina is not perturbed. In wild-type retinas, phosphohistone H^3^-positive mitotic cells are localised to the outer surface of the retina whereas Brn3a-positive RGCs are restricted to the RGC layer at the inner surface of the retina (Fig. 6A). Both cell types are distributed identically in *robo2*-deficient retinas (Fig. 6B). Other markers of differentiated retinal cells (Islet 1/2: RGC and amacrine cells; Pax6: RGC and amacrine cells; AP2α: amacrine cells) also gave identical labelling patterns in wild-type and *robo2*-deficient retinas (Fig. 6C–H). Gross defects in retinal organisation therefore are unlikely to underlie the intraretinal guidance errors that occur in the *robo2*-deficient mice.

## Discussion

Robo2 and, to a lesser extent, Robo1 have been shown previously to be key regulators of RGC axon guidance at the optic chiasm and optic tract (Plachez et al., 2008). Here we expand these findings and demonstrate clearly that Robo2 is required for the first step in RGC axon guidance, the directed growth of the axons within the retina to exit the eye. Together with previous work (Fricke et al., 2001; Hutson and Chien, 2002; Plachez et al., 2008), these findings provide direct evidence that Robo2 is the predominant Robo required for RGC axon pathfinding and, like its Slit ligands, is essential for RGC axon guidance throughout the entire optic pathway (Plump et al., 2002; Thompson et al., 2006a,b; Plachez et al., 2008).

### Robo2 mediates the distinct guidance activities of Slits within dorsal and ventral retina

A surprising feature of Slit signalling within the retina is that it controls distinct guidance events within dorsal and ventral retina (Thompson et al., 2006a). Within dorsal retina exclusively, Slit2 controls the initial polarity of RGC axon extension. By contrast, in ventral retina predominately, a combination of Slit1 and Sit2 helps restrict the RGC axons to their correct location within the retina, the OFL (Thompson et al., 2006a). This is despite the fact that both *slit1* and *slit2* are expressed throughout the retina (Erskine et al., 2000; Niclou et al., 2000; Ringstedt et al., 2000; Thompson et al., 2006a) and, in vitro, dorsal and ventral RGC axons respond similarly to Slits (Erskine et al., 2000; Thompson et al., 2006a). In the *Drosophila* ventral nerve cord, differential function and expression of Robo receptors underlie the guidance of commissural axons across the midline and their subsequent selection of distinct longitudinal pathways (Rajagopalan et al., 2000a,b; Simpson et al., 2000a,b). This raises the intriguing possibility that the different functions of Slits within the vertebrate retina are mediated via distinct receptors. However, this does not appear to be the case. Our expression analyses have confirmed that Robo1 and Robo2 are the only known Slit receptors expressed by RGCs. Additionally, we and others have detected no obvious difference in the expression of Robo1 or Robo2 within dorsal and ventral retina (Erskine et al., 2000; Niclou et al., 2000; Ringstedt et al., 2000; Plachez et al., 2008). Finally, although Robo1 is expressed by a subset of RGCs, suggesting a potential role in the differential guidance of these cells, our analyses of mice lacking *robo1* or *robo2* have demonstrated clearly that Robo2 alone is sufficient for both aspects of Slit-mediated intraretinal axon pathfinding. In the absence of Robo1, Robo2 mRNA and protein expression is not altered in the nervous system (Long et al., 2004; López-Bendito et al., 2007), arguing against a compensatory role for Robo2 in the *robo1* mutants. Redundant interactions between Robo1 and Robo2 also are unlikely as the intraretinal guidance errors in the *robo2* and *slit* mutants are qualitatively and quantitatively identical. Instead, the simplest explanation for the lack of intraretinal guidance errors in *robo1* mutants is the relatively late onset of *robo1* expression within the retina. *robo1* cannot be detected within the retina until at least 2 days later than *robo2* (Erskine et al., 2000), suggesting that Robo1 may not be present on RGC axons as they navigate through the eye. In support of this, a requirement for Robo1 in later stages of RGC axon pathfinding, at the optic chiasm and tract, has been demonstrated (Plachez et al., 2008). Thus, the differential timing of Robo1 and Robo2 expression in RGCs, combined with the phenotype of the *robo1*- and *robo2*-deficient mice, suggests strongly that Robo2 is the only Robo required for intraretinal axon pathfinding.

Patterning of the retina along its dorsal–ventral and nasal–retina axes is a fundamental aspect of vertebrate eye development. Along the dorsal–ventral axis, the restricted expression of transcription factors such as *Tbx5* and *Vax2* is essential for the specification of dorsal and ventral characteristics respectively (Koshiba-Takeuchi et al., 2000; Sakuta et al., 2001). This includes the graded expression of guidance molecules, such as EphBs/ephrinBs, essential for the topographic mapping of RGC axons in visual targets (Schulte et al., 1999; Koshiba-Takeuchi et al., 2000). It is therefore not entirely unexpected that distinct guidance mechanisms direct intraretinal axon pathfinding within these different regions of the retina. Indeed several molecules have been identified, including EphBs and BMP Receptor 1b, that, due to their restricted expression within dorsal or ventral retina, are required exclusively for the intraretinal guidance of dorsal or ventral RGC axons (Birgbauer et al., 2000, 2001; Liu et al., 2003). Thus, the simplest explanation for the distinct requirements of Slit–Robo signalling in dorsal and ventral retina is that this reflects redundancy with other guidance signals differentially expressed within these regions of the retina. A key challenge for the future will be to identify the molecular nature of these signals.

### Slit–Robo signalling is not required for the correct targeting of RGC axons within the contralateral eye

In wild-type animals, after crossing at the optic chiasm, a small proportion of RGC axons innervate the contralateral eye rather than entering the optic tract. Whether these retina–retina axons serve any function, for example providing a substrate for the guidance of isthmo-optic axons (Thanos, 1999) or are the result of guidance errors is not known. In support of the later suggestion, in mice lacking *slit1/2* or *robo2*, the proportion of axons innervating the contralateral eye is increased substantially, demonstrating an important role for Slit–Robo signalling in preventing growth in this direction (Plump et al., 2002; Plachez et al., 2008). A similar increase in retina–retina projections is also found in mice lacking specific heparan sulphate synthesising enzymes (Inatani et al., 2003; Pratt et al., 2006), most likely reflecting the role of heparan sulphate proteoglycans in augmenting Slit–Robo interactions (Hussain et al., 2006; Piper et al., 2006).

In wild-type animals, the retina–retina projecting axons terminate predominately in the ventral region of the contralateral retina. Given the increased proportion of RGC axons that innervate the contralateral eye in the absence of Slit1/2 or Robo2 (Plump et al., 2002; Plachez et al., 2008) and their ventral bias, one explanation for the aberrant axons located within the outer layers of *slit1/2*- or *robo2*-deficient retinas, including their predominate ventral location (Thompson et al., 2006a), is that these are aberrant retina–retina axons. However, this is not the case. In the vast majority of cases, in mice lacking Slit–Robo signalling, RGC axons innervating the contralateral eye grow normally into the OFL. In only 1/6 retinas did a small number of retina–retina axons grow aberrantly within the outer layers of the retina. This demonstrates clearly that, in the absence of Slit–Robo signalling, the ectopic presence of axons within the outer layers of the retina is mainly the result of guidance errors made by RGC axons prior to exiting the retina rather than targeting defects.

### Slit–Robo signalling controls the initial polarity of RGC axon outgrowth but is not essential for growth towards the optic disc or exit from the eye

A key feature of intraretinal axon guidance is the highly directed and organised manner in which the RGC axons project towards the optic disc (reviewed by Erskine and Herrera, 2007; Bao, 2008; Erskine and Thompson, 2008). This is achieved by the differential expression and function of a range of different guidance signals along the central–peripheral axis of the retina. Some factors, such as netrin and EphBs are required exclusively within the central region of the retina for the targeting of RGC axons to the optic disc and their exit from the eye (Deiner et al., 1997; Höpker et al., 1999; Birgbauer et al., 2000). Other factors, such as Sonic hedgehog and inhibitory guidance cues under the control of the transcription factor *Zic3*, are expressed more dynamically in gradients across the retina and are essential for the normal central–peripheral extension of the RGC axons (Zhang et al., 2004; Kolpak et al., 2005). Selective fasciculation mediated by a range of different cell adhesion molecules also plays an important role in the directed extension of RGC axons towards the optic disc (Pollerberg and Beck-Sickinger, 1993; Brittis et al., 1995; Ott et al., 1998; Monnier et al., 2001; Avci et al., 2004; Weiner et al., 2004; Zelina et al., 2005). Previously we have shown that Slit2, possibly originating from the lens, is a key factor controlling the initial polarity of RGC axon extension from recently differentiated cells located within the retinal periphery (Thompson et al., 2006a). Here we show that this function of Slits is mediated via Robo2. Furthermore, by analysing the trajectory of small groups of RGC axons in detail, we provide direct evidence that, once axons reach the OFL, Slit–Robo signalling is required exclusively for guidance within the retinal periphery and not more central regions of the retina or exit from the eye. This highlights the step-wise, combinatorial nature of RGC axon guidance along the central–peripheral axis of the retina. First, factors such as Slits and Robos control the initial direction of RGC axon extension (Thompson et al., 2006a). Thereafter, other signals take over to drive growth towards the optic disc and out of the eye. Thus, even within this relatively simple system, integrated interactions between multitudes of differentially expressed guidance signals are essential for normal pathfinding to occur.

## Figures and Tables

**Table 1:** Number of wild-type and *robo2*-deficient embryos with retina–retina axons located in the outer layers of the contralateral retina.

Genotype	Number analysed	Number with axons in outer retina
WT	2	0
*robo2^+/−^*	12	0
*robo2^−/−^*	6	1 (3 axon bundles)
